# The humanistic and economic burden of treatment-resistant depression in Europe: a cross-sectional study

**DOI:** 10.1186/s12888-019-2222-4

**Published:** 2019-08-07

**Authors:** Dena H. Jaffe, Benoit Rive, Tom R. Denee

**Affiliations:** 1 Kantar, 4 Ariel Sharon St., Givatayim, 53447 Tel Aviv, Israel; 2 Janssen-Cilag BV, Paris, France; 3 Janssen-Cilag Limited, High Wycombe, UK

**Keywords:** Activity impairment, Healthcare resource use, Health-related quality of life, Work impairment, Treatment -resistant depression

## Abstract

**Background:**

A patient is considered to suffer from treatment resistant depression (TRD) when consecutive treatment with two products of different pharmacological classes, used for a sufficient length of time at an adequate dose, fail to induce a clinically meaningful effect (inadequate response). The primary aim of the current study was to examine the humanistic and economic burden of TRD in five European countries, France, Germany, Italy, Spain and the United Kingdom, by comparing with non-treatment resistant depression (nTRD) and general population respondents.

**Methods:**

The sample for this retrospective observational study was taken from the 2017 National Health and Wellness Survey conducted in five European countries. Demographic and patient characteristics were examined for TRD patients compared to respondents with nTRD and to the general population using chi-square tests or one-way analysis of variance for categorical or continuous variables, respectively. Generalized linear models were performed to examine group differences after adjusting these estimates for confounders.

**Results:**

A total 52,060 survey respondents were examined, of which 2686 and 622 were considered to have non-treatment resistant and treatment-resistant depression, respectively. Relative to the general population, nTRD and TRD respondents reported significant decrements in health-related quality of life, including lower adjusted mental (− 12.1 vs. -18.1) and physical (− 2.5 vs. -5.4) component scores of the SF-12v2 and increased adjusted relative risk for work (2.2 vs. 2.7) and activity (1.9 vs. 2.5) impairment (all *p* < 0.001). Additionally, healthcare resource utilization was significantly higher for TRD patients more so than nTRD, compared to the general population, especially for healthcare professional visits (odds ratio nTRD = 5.4; TRD = 15.9, *p* < 0.001).

**Conclusions:**

In conclusion, TRD patients had significantly lower quality of life, greater work productivity and activity impairment, and increased healthcare resource utilization as compared with nTRD and general population. The study findings suggest an unmet need exists among TRD patients in Europe.

**Electronic supplementary material:**

The online version of this article (10.1186/s12888-019-2222-4) contains supplementary material, which is available to authorized users.

## Background

Treatment-resistant depression (TRD) is defined as major depressive disorder (MDD) in adults who have not responded to at least two different antidepressant treatments in the current moderate to severe depressive episode. Treatment resistance occurs commonly in up to 30% of the treated MDD patient population [1]. Specifically, Rush et al. has shown that remission rates among MDD patients following their first or second treatment course is 37 and 31% respectively [2]. MDD is a psychiatric disorder diagnosed through a clinical assessment against criteria set out in the 5th edition of Diagnostic and Statistics Manual for Mental Disorders [3]. MDD is a debilitating psychiatric condition occurring when an individual continuously experiences a combination of five or more different symptoms, especially depressed mood and/or a loss of interest in daily activities, for at least 1 week; these symptoms result in clinically meaningful distress and/or functional impairment in important life domains, such as employment and interpersonal relationships [4].

The level of functional impairment associated with depression has been shown to equal or exceed that associated with other severe chronic general medical conditions, such as diabetes and congestive heart failure [4]. The lifetime prevalence of MDD was estimated at 12.8% in the European Study of the Epidemiology of Mental Disorders, making it the most common mental health condition in the European Union [5]. Furthermore, in the European Union, it is estimated that 6.9% of the total population aged over 14 years, experience depression during a 12-month period [6]. According to the World Mental Health surveys, sociodemographic characteristics such as female sex, younger age, lower education, high teen childbearing, marital problems (separated or divorced), unstable employment, reduced role functioning, persistence and severity of secondary disorders were consistently associated with a higher risk of MDD [7].

Recommended first-line treatment for MDD includes antidepressant medications, psychological-behavioral therapies, or a combination of the two approaches [8]. However, research suggests that up to two-thirds of patients do not respond to their initial treatment with an antidepressant [9]. Additionally, a prospective multicenter European study reported that over a quarter of patients with MDD did not respond after three antidepressant treatments [10].

Despite advances in the treatment of MDD, only 40–70% of patients respond to the treatment whereas an estimated 10–30% of patients exhibit TRD [1, 11]. Furthermore, there is no universally accepted definition of TRD, which has been treated as a homogenous entity [12–14]. However, the European Medicines Agency and the Food & Drug Administration define TRD as MDD that has been treated with two antidepressant products of different pharmacological classes, used for a sufficient length of time at an adequate dose, and failed to show an adequate response [15]. In addition, this definition has also been used in some prior studies [9, 16]. No treatment has been approved for TRD in Europe so far.

TRD was shown to be associated with comorbid anxiety disorder, higher suicide risk, melancholic features, and a lack of response to an initial antidepressant [17], as well as a body mass index (BMI) ≥30 kg/m^2^, a depressive episode lasting > 2 months, being in psychotherapy, sexual dysfunction, and greater depression severity [18].

Previous research suggests that TRD results in a substantial burden on both patients and society above that posed by MDD alone. A systematic review conducted by Mrazek and colleagues [19] demonstrated the negative impact of TRD on health-related quality of life (HRQoL), with self-ratings between 0.26 to 0.41 points lower (on a scale of 0–1) for adults with TRD, relative to those with MDD in remission or who achieved a response to treatment. A US study reported that patients with TRD (MDD-treated patients with depressive symptoms patient health questionnaire ≥15 had significantly higher comorbidity burden, lower HRQoL, and impaired work and activity compared to those with MDD-treated patients with minimal depressive symptoms [20].

TRD imposes a substantial burden on healthcare resource utilization (HRU). Few studies showed that TRD patients had double the hospitalization rate and more outpatient and emergency room (ER) visits compared with non-TRD (nTRD) and non-MDD patients from the general population [20, 21]. In addition, the annual direct medical costs for healthcare and indirect costs due to productivity loss were $5481 and $4048 higher, respectively, among adults with TRD than for MDD remitters and responders [19]. Moreover, studies in the US and Brazil have reported that the overall healthcare costs of TRD was nearly twice or more than twice as high as those without TRD [22–25]. However, there is a paucity of recent research that assesses the burden of TRD, in general, and in Europe, specifically. Given the high prevalence of MDD in the general adult population within Europe, as well as the frequency of TRD among those with MDD, a better understanding of the burden of TRD in this region is necessary.

The primary aim of the current study was to examine the humanistic and economic burden of TRD in five European countries by comparing with nTRD and general population respondents.

## Methods

### Sample

The sample for this retrospective observational study was taken from the 2017 National Health and Wellness Survey (NHWS; Kantar Health, New York, USA) that was conducted in five European countries: France, Germany, Italy, Spain, and the United Kingdom (UK). The NHWS, which is part of the Kantar patient-centered research program (PaCeR) and is not publicly available, is self-administered general health survey of the adult population (aged ≥18 years) administered via the Internet and designed to reflect the health in the general population of each country. The European NHWS was reviewed and granted exemption status by the Pearl Institutional Review Board (Indianapolis, IN; 17-KANT-141). All respondents provided informed consent.

**Fig. 1:**
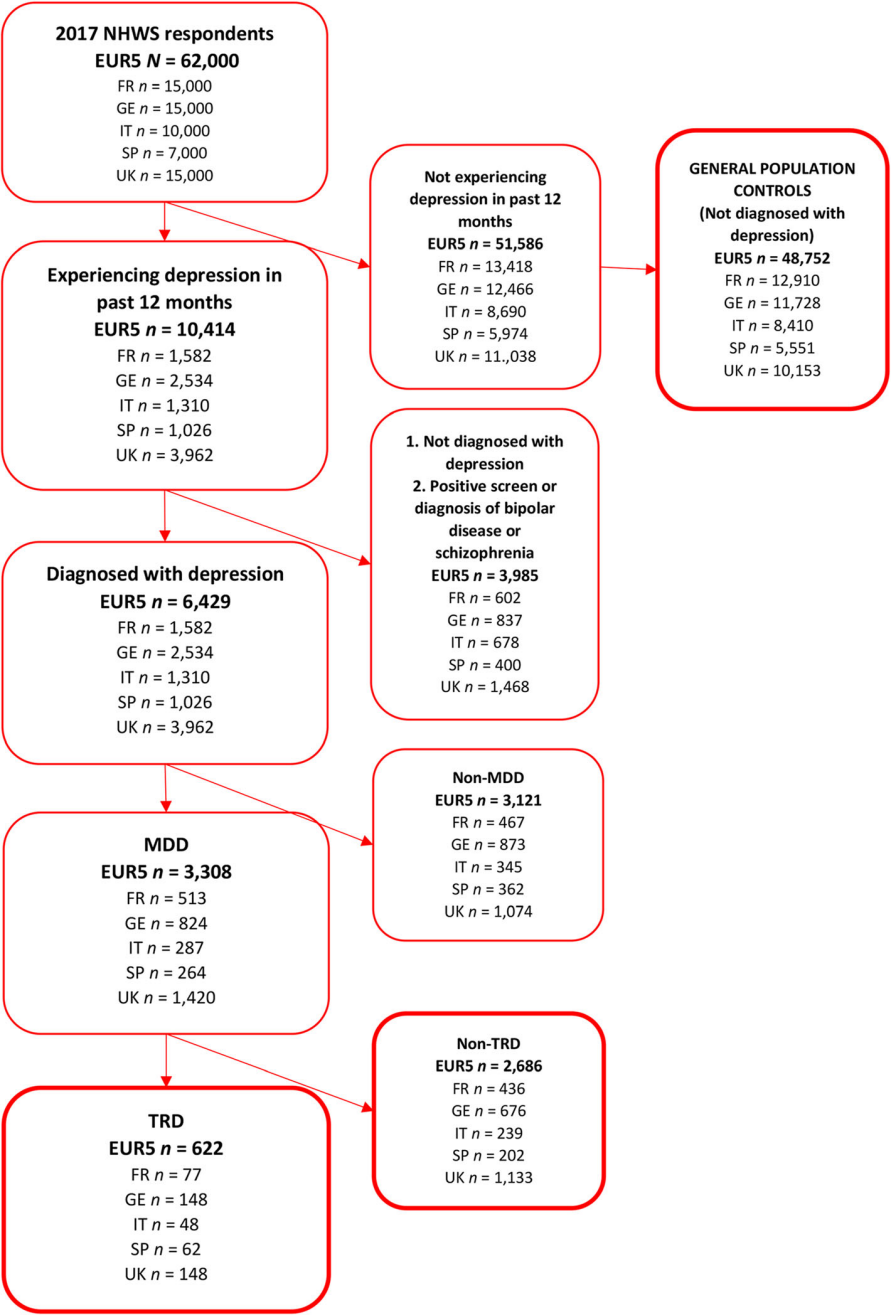
Study flow chart – cohort identification. *FR* France, *GE* Germany, *IT* Italy, *MDD* major depressive disorder, *SP* Spain, *TRD* treatment-resistant depression, *nTRD* non- treatment-resistant depression, *UK* United Kingdom

Patients with MDD were classified as those who experienced depression in the past 12 months, received a diagnosis for depression, and indicated that they were currently treated with ≥1 medication for depression. Excluded were patients with a co-existing condition of bipolar disease based on self-reported diagnosis or a positive screen on the Mood Disorder Questionnaire [26] or a self-reported diagnosis of schizophrenia. Potential patients with TRD were identified as patients fulfilling at least one of the following three criteria: (1) currently prescribed ≥2 medications for depression for ≥3 months, (2) currently prescribed monotherapy with a Monoamine Oxidase Inhibitor (MAOI) or Reversible Inhibitor of Monoamine Oxidase (RIMA) (prescribed only in France, Germany and the UK), and (3) a depression symptom score of ≥10 using the Patient Health Questionnaire (PHQ-9). The PHQ-9 is a validated scale used to screen for depression and assess severity with scores ranging from 0 to 27 with scores at or above 10 indicating symptoms of moderate to severe depression [27]. The study sample compared MDD patients with TRD to (a) MDD patients without treatment resistance (nTRD) and to (b) the general population who did not experience depression in the past 12 months nor have a diagnosis of depression (Fig. 1).

### Measures

#### Depression-specific measures

Depression-specific variables examined included family history of depression (yes vs. no), length of diagnosis (calculated by year of diagnosis, relative to year of the survey; < 1 year vs. 1–3 years vs. 3–5 years vs. > 5 years), participation in talk therapy (yes vs. no), anxiety, suicide ideation and current classes of MDD medications. The presence of suicide ideation was assessed using the PHQ-9 question, “Over the past 2 weeks, how often have you been bothered by thoughts that you would be better off dead or of hurting yourself in some way?”

#### Demographics and health characteristics

The demographic variables examined included age, sex, marital status (married/living with a partner vs. not married), education (university degree vs. less than a university degree), and annual household income (low < 20,000 € or £, medium 20,000–39,000 € or £, high 40,000+ € or £, or decline to answer). For health characteristics, NHWS respondents provided data on BMI, smoking status, alcohol use, and exercise behavior. All information was collected according to country residence.

#### Comorbidities

The burden of comorbidities was measured using the Charlson Comorbidity Index (CCI) [28]. Additionally, specific physical comorbidities were assessed, which included a self-reported diagnosis of anemia, arrhythmia, cancer, chronic heart failure, chronic kidney disease, diabetes (type 1 or 2), hepatitis (A, B, or C), hypertension, and rheumatoid arthritis.

#### Health-related quality of life

The EuroQol-5 Dimensions 5-levels (EQ-5D-5 L) was used to assess HRQoL. The EQ-5D-5 L is a self-reported measure of health for clinical and economic appraisal [29] consisting of five questions regarding mobility, self-care, usual activities, pain/discomfort, and anxiety/depression and a visual analogue scale (EQ-VAS). The EQ-VAS asks respondents to indicate on a line their self-rated health, with the endpoints being “Best imaginable health state” and “Worst imaginable health state”.

The Medical Outcomes Study 12-Item Short Form Survey Instrument version 2 (SF-12v2), which is a multipurpose, generic health status instrument consisting of 12 questions was also used to measure HRQoL [30]. Eight health domains, physical functioning, physical role limitations, bodily pain, general health, vitality, social functioning, emotional role limitations, and mental health, were calculated as well as two summary scores, the physical component summary (PCS) and mental component summary (MCS), each normalized to a mean of 50 and a standard deviation (SD) of 10 for the general population of the US. Higher scores indicate better health status.

#### Work productivity loss and activity impairment

Work productivity loss among employed respondents and activity impairment among all respondents in the past week was assessed using the six-item Work Productivity and Activity Impairment-General Health (WPAI-GH) questionnaire [31]. The WPAI-GH assesses presenteeism (reduced productivity while at work), absenteeism (time absent from work), overall work productivity impairment (a combination of presenteeism and absenteeism), and activity impairment. Scores on the WPAI-GH represent the percentage of time impaired in the past week.

#### Healthcare resource utilization

Participants were asked to provide the number of each type of HRU (healthcare professional [HCP] visits, ER visits, hospitalizations, other types of visits, psychiatrist visits, and psychologist/therapist visits). These responses were then converted into a categorical variable representing the percentage who reported ≥1 event and those who reported none, for each type of event.

### Statistical analysis

Demographic and patient characteristics were examined for TRD patients compared to respondents with nTRD and to the general population using chi-square tests or one-way analysis of variance for categorical or continuous variables, respectively. All socio-demographic and health status variables were included in models. Generalized linear models were performed to examine group differences after adjusting these estimates for confounders. For normally distributed outcome variables, a normal distribution and identity function were specified, whereas a negative binomial distribution and log-link function were specified for positively skewed outcome variables. For the generalized linear models, two-sided *p*-values < 0.05 were considered to be statistically significant. Estimated marginal means and 95% confidence intervals (CI) were calculated for each group on all outcomes as well as adjusted difference, relative risks (RR), or odds ratios (OR) and 95% CI for TRD and nTRD versus the general population.

## Results

Of the total 52,060 respondents, 3308 had MDD (France = 513, Germany = 824, Italy = 287, Spain = 264, and UK = 1420) and were included in the study (Additional file 4: Figure S1), with 18.8% defined as TRD. The comparison of baseline demographic data and clinical characteristics between TRD, nTRD, and general population groups are shown in Table 1. Individuals with MDD (TRD and nTRD subsets combined) were generally younger than that of general population and were disproportionately female; they also were less likely to have a university degree or be married or living with partner. Respondents from the general population exercised more, were fewer non-smokers, and had lower CCI compared with MDD respondents. Within the MDD population, TRD patients had significantly more cases of anemia (*p* < 0.001), chronic heart failure (*p* = 0.020), and rheumatoid arthritis (*p* = 0.013) than nTRD patients. Comorbidities like arrhythmia (*p* = 0.004), chronic kidney disease (*p* = 0.031), anemia, chronic heart failure, diabetes, hypertension, and rheumatoid arthritis (all *p* < 0.001) more commonly occurred in TRD and nTRD patients than the general population (Table 1).

**Table 1:** Demographic and clinical characteristics among TRD, nTRD, and general population groups

Variable	(a) TRD (*n* = 622)		(b) nTRD (*n* = 2686)		(c) General population (*n* = 48,752)		*P-*value		
	%	N	%	N	%	N	a vs. b	a vs. c	b vs. c
Sex									
Male	32.3%	201	35.0%	941	47.4%	23,095	0.199	<0.001	<0.001
Female	67.7%	421	65.0%	1745	52.6%	25,657			
Age, years									
18 to 29	13.3%	83	14.0%	376	16.7%	8140	0.001	<0.001	<0.001
30 to 39	12.5%	78	14.6%	391	15.7%	7652			
40 to 49	26.4%	164	22.7%	611	17.3%	8449			
50 to 59	29.7%	185	24.6%	661	16.3%	7926			
60+	18.0%	112	24.1%	647	34.0%	16,585			
Country									
France	12.4%	77	16.2%	436	26.5%	12,910	0.020	<0.001	<0.001
Germany	23.8%	148	25.2%	676	24.1%	11,728			
Italy	7.7%	48	8.9%	239	17.3%	8410			
Spain	10.0%	62	7.5%	202	11.4%	5551			
UK	46.1%	287	42.2%	1133	20.8%	10,153			
Marital Status (married or living with partner)^a^	49.8%	310	50.9%	1366	62.7%	30,561	0.890	<0.001	<0.001
Education (completed university)^b^	26.2%	163	28.9%	777	38.3%	18,673	0.182	<0.001	<0.001
Household Income									
Low (< 20,000 € or £)	38.6%	240	37.1%	996	23.1%	11,257	0.818	<0.001	<0.001
Medium (≥20,000- < 40,000 € or £)	33.3%	207	33.6%	902	37.5%	18,281			
High (≥40,000 € or £)	20.3%	126	21.8%	585	29.1%	14,180			
Decline to answer	7.9%	49	7.6%	203	10.3%	5034			
Employed	39.2%	244	49.7%	1334	55.3%	26,951	<0.001	<0.001	<0.001
CCI (mean, SD)	0.76	1.22	0.57	1.05	0.29	0.77	<0.001	<0.001	<0.001
Body Mass Index									
Underweight (<18.5 kg/m2)	4.3%	27	3.3%	88	3.3%	1617	0.001	<0.001	<0.001
Normal weight (≥18.5 to <25.0 kg/m2)	25.4%	158	31.6%	848	43.7%	21,328			
Overweight (≥25.0 to <30.0 kg/m2)	27.3%	170	30.1%	808	31.4%	15,293			
Obese (≥30.0 kg/m2)	32.0%	199	26.0%	698	14.9%	7243			
Decline to Answer	10.9%	68	9.1%	244	6.7%	3271			
Exercised	39.4%	245	51.0%	1370	62.2%	30,342	<0.001	<0.001	<0.001
Alcohol use (daily)	5.5%	34	5.8%	155	8.0%	3881	0.827	<0.001	<0.001
Current smoker	32.3%	201	32.0%	859	22.4%	10,900	0.714	<0.001	<0.001
Comorbidities									
Anemia	10.8%	67	5.9%	159	2.5%	1202	<0.001	<0.001	<0.001
Arrhythmia	4.0%	25	3.2%	85	2.3%	1107	0.284	0.004	0.003
Cancer	5.8%	36	6.5%	174	5.2%	2552	0.525	0.539	0.005
Chronic heart failure	3.1%	19	1.6%	44	0.9%	434	0.020	<0.001	<0.001
Chronic kidney disease	1.4%	9	1.3%	36	0.7%	347	0.836	0.031	<0.001
Diabetes	12.9%	80	11.7%	313	7.3%	3579	0.401	<0.001	<0.001
Hepatitis A	0.8%	5	1.1%	29	0.9%	421	0.539	0.873	0.242
Hypertension	29.4%	183	26.1%	702	19.1%	9302	0.095	<0.001	<0.001
Rheumatoid arthritis	7.4%	46	4.9%	132	2.2%	1050	0.013	<0.001	<0.001

*CCI* Charlson comorbidity index, *nTRD* non-treatment-resistant depression, *TRD* treatment-resistant depression

^a^< 0.3% had missing values

^b^Approximately 1% declined to answer

Depression-related characteristics varied within MDD patients (Table 2). TRD patients were observed to have a longer average time since depression diagnosis than nTRD patients (12.9 ± 10.2 years vs 10.4 ± 10.0 years, *p* < 0.001). TRD patients also more frequently reported family history of depression, anxiety, and suicide ideation (all *p* < 0.001). Compared with nTRD patients, TRD patients had significantly higher rates of usage of other depression medication like tetracyclic antidepressants/tricyclic antidepressants (21.9%), potential augmentation/combination medications such as benzodiazepine (11.3%), anti-psychotics (8.0%), norepinephrine–dopamine reuptake inhibitors/norepinephrine reuptake inhibitors (2.9%), and MOAI/RIMA (1.0%; all *p* < 0.001).

**Table 2:** Depression-related characteristics among respondents with TRD or nTRD

Parameters	TRD (*n* = 622)		nTRD (*n* = 2686)		*P-*value
	%	N	%	N	
Family history of depression	47.7%	297	38.2%	1026	<0.001
Participation in talk therapy	33.3%	207	22.9%	616	<0.001
Anxiety	78.0%	485	61.6%	1654	<0.001
Suicide ideation	17.0%	106	8.2%	220	<0.001
Prescription medication use for depression					
SSRI/SNRI/SARI/NaSSA	92.6%	576	90.0%	2417	<0.001
TeCA/TCA	21.9%	136	9.7%	261	<0.001
Benzodiazepine	11.3%	70	1.2%	32	<0.001
Anti-Psychotics	8.0%	50	1.0%	26	<0.001
NDRI/NRI	2.9%	18	0.9%	25	<0.001
MOAI/RIMA	1.0%	6	.0%	1	<0.001

*MAOI* Monoamine oxidase inhibitors, *MDD* major depressive disorder, *nTRD* non-treatment resistant depression, *NA* not applicable, *NaSSA* Noradrenergic and specific serotonergic antidepressant, *NDRI* Norepinephrine–dopamine reuptake inhibitors, *NRI* norepinephrine reuptake inhibitors, *RIMA* Reversible inhibitors of monoamine oxidase A, *SD* standard deviation, *SSRI* Selective serotonin reuptake inhibitors, *SNRI* Serotonin–norepinephrine reuptake inhibitors, *TCA* tricyclic antidepressants, *TeCA* tetracyclic antidepressants, *TRD* treatment resistant depression

### Health-related quality of life

Table 3 provides the adjusted differences for HRQoL among respondents with TRD and nTRD compared to general population controls. After adjusting for demographics and health characteristics, TRD and nTRD groups had poorer health status as assessed by the EQ-5D-5 L (− 0.329 and − 0.173, respectively, both *p* < 0.001) and EQ-VAS (− 27.440 and − 16.724, respectively, both *p* < 0.001) compared to the general population. TRD patients when compared to general population, reported significantly lower HRQoL scores in all measures including MCS (− 18.145) and PCS (− 5.362). Significantly lower scores at *p* < 0.001 were noted for all subdomains of the SF-12 for TRD versus general population respondents: bodily pain (− 8.771), general health (− 11.414), mental health (− 15.781), physical functioning (− 7.104), emotional role functioning (− 17.510), physical role functioning (− 9.523), social functioning (− 14.963) and vitality (− 11.458). Similar findings with the same trend in HRQoL were observed across all the European countries (Additional file 1: Table S1).

**Table 3:** Adjusted differences for HRQoL among respondents with TRD or nTRD compared to general population controls

Parameters	General population controls versus	
	TRD^a^	nTRD^a^
	Adjusted difference (LCL to UCL)	Adjusted difference (LCL to UCL)
EQ-5D Index	−0.329 (−0.343 to −0.315)	−0.173 (−0.180 to −0.166)
Health Status, EQ VAS	−27.440 (−28.981 to −25.900)	−16.724 (−17.489 to −15.959)
MCS	−18.145 (−18.810 to −17.479)	−12.151 (−12.481 to −11.820)
PCS	−5.362 (−5.978 to −4.745)	−2.507 (− 2.813 to − 2.201)
SF-12 Bodily Pain	−8.771 (−9.503 to −8.039)	− 5.142 (− 5.506 to − 4.779)
SF-12 General Health	−11.414 (− 12.053 to −10.775)	−6.659 (−6.976 to −6.342)
SF-12 Mental Health	−15.781 (− 16.441 to − 15.121)	−10.473 (− 10.800 to − 10.145)
SF-12 Physical Functioning	−7.104 (−7.743 to − 6.465)	−3.762 (− 4.080 to −3.445)
SF-12 Role Emotional	−17.510 (− 18.299 to − 16.722)	− 11.374 (− 11.766 to − 10.983)
SF-12 Role Physical	−9.523 (− 10.198 to − 8.848)	− 5.619 (− 5.954 to − 5.284)
SF-12 Social Functioning	−14.963 (− 15.635 to − 14.290)	− 9.532 (− 9.866 to − 9.198)
SF-12 Vitality	−11.458 (− 12.149 to − 10.767)	− 7.335 (− 7.678 to − 6.992)

*EQ-5D* EuroQol-5D, *EQ VAS* EuroQol Visual analogue scale, *HRQoL* health-related quality of life, *LCL* lower confidence interval, *MCS* mental component summary score, *nTRD* non-treatment resistant depression, *PCS* physical component summary score, *SD* standard deviation, *SF-12* Medical Outcomes Study 12-Item Short Form Survey Instrument version 2, *TRD* treatment resistant depression, *UCL* upper confidence interval

^a^Generalized linear models were used adjusted for sociodemographic and health status variables. Comparisons versus the general population were statistically significant at *p* ≤ 0.001

### Work productivity loss and activity impairment

**Fig. 2:**
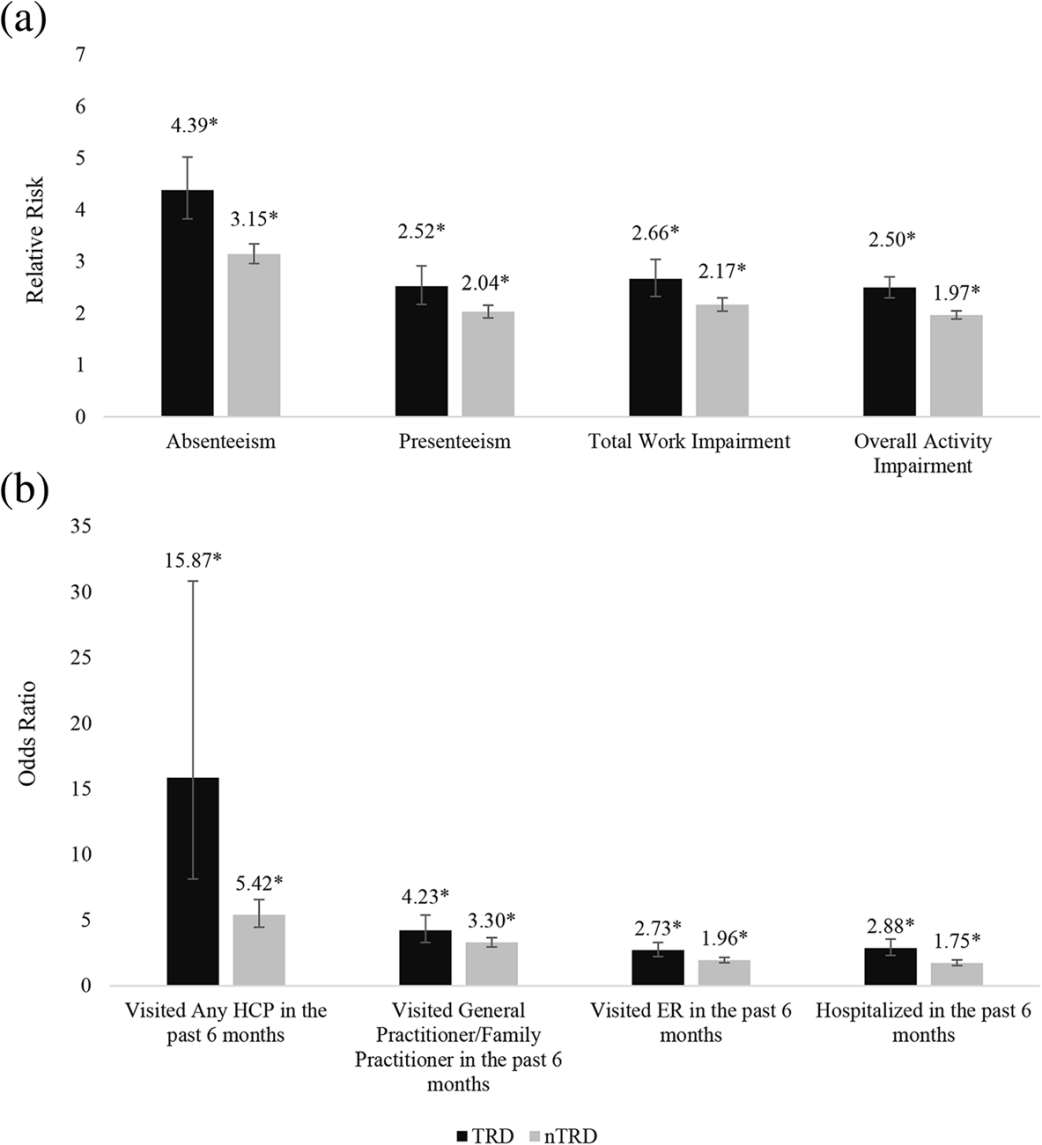
**a** WPAI and (**b**) HRU outcomes among TRD, nTRD respondents vs. general population ^a^. *HCP* healthcare professional, *ER* emergency room, *TRD* treatment-resistant depression, *nTRD* non- treatment-resistant depression. ^a^ Generalized linear models were used adjusted for sociodemographic and health status variables. * *p* < 0.001

TRD patients had significantly higher absenteeism (adjusted RR: 1.53; 95% CI 1.32 to 1.77, *p* < 0.001), presenteeism (adjusted RR: 1.29; 95% CI 1.09 to 1.51, *p* = 0.002), greater overall work impairment (adjusted RR: 1.29; 95% CI 1.12 to 1.50, *p* = 0.001) and activity impairment (adjusted RR: 1.28; 95% CI 1.67 to 1.39, *p* < 0.001) than the nTRD population (Fig. 2; for all *p* < 0.001), with even larger adjusted RR compared to the general population. A similar degree of work productivity loss and activity impairment was observed across the five European countries for TRD patients when compared to nTRD patients (Additional file 2: Table S2). Similarly, in TRD versus general population, all five European countries reported similar rates of work productivity loss and activity impairment (Additional file 2: Table S2).

### Healthcare resource utilization

When compared to nTRD patients, the odds of having visited an HCP was significantly higher in TRD patients (adjusted OR: 2.98; 95% CI 1.48, 6.00, *p* = 0.002). TRD patients also had higher odds of visiting a general/family practitioner (adjusted OR: 1.29; 95% CI 0.98, 1.67, *p* = 0.063), higher odds of hospitalization (adjusted OR: 1.79; 95% CI 1.41, 2.28, *p* < 0.001), and higher odds of ER visits (adjusted OR: 1.53; 95% CI 1.23, 1.91, *p* < 0.001) in the past six months (Fig. 2). In general, the HRU was significantly and consistently higher among TRD than nTRD patients across all five European countries (Additional file 3: Table S3). Relative to the general population, TRD patients had significantly higher number of general/family practitioner visits, hospitalization and ER visits in the past six months (Fig. 2; for all *p* < 0.001). Overall, across all the five European countries, TRD patients exhibited highest HCP visits in the past six months (Additional file 3: Table S3).

## Discussion

The current study is the first multi-national study to report the burden of TRD by assessing the HRQoL, work productivity and activity impairment, and HRU across five European countries. We identified 3308 MDD patients with 18.8% defined as TRD. The demographic characteristics in our study were found to be consistent with previous studies [19, 20, 32, 33] with majority of MDD patients being women who were separated or divorced, and in their mid-40s. Differences within MDD subgroups were not apparent by sex, smoking or alcohol use, although distributional differences by age group may suggest an area for targeted interventions.

In the current study, the prevalence of physical comorbidities, such as anemia, chronic heart failure, diabetes, hypertension and rheumatoid arthritis, among MDD patients was higher compared to the general population, although the temporal relation of this association is unclear. Additionally, our study showed that within the MDD population, respondents with TRD have different comorbid profiles. Findings of greater mental health-related issues, such as anxiety and suicidal ideation among the TRD group compared to the nTRD group is in line with a previous study reported by Russell et al. [34].

In the present study, TRD patients had worse HRQoL defined by the EQ-5D and SF-12v2, higher work productivity loss, and increased healthcare use, compared with nTRD respondents and general population, even after adjusting for potential confounders. TRD patients consistently reported greater impairments in HRQoL, with lower MCS, PCS, SF-12v2, EQ-5D-5 L, and EQ-VAS scores. In a systematic review, TRD was associated with poorer mental health and physical functioning, as measured by Medical Outcomes Study 36-Item Short Form Survey Instrument (SF-36) MCS and PCS scores [19]. Similar findings were reported in another systematic review by Johnston et al. (2018), in which increasing levels of TRD/non-response correlated with reduced HRQoL and health status as assessed by the SF-36 or SF-12 health domain and EQ-5D utility scores [35].

In the current study, work-related productivity losses were significantly greater among TRD patients than for nTRD or general population respondents. The relative risk of absenteeism was highest for TRD respondents with a 3–4 times greater risk. TRD respondents had about double the risk of presenteeism, total work and overall activity impairments compared to the general population. While there were data from studies conducted in Europe and US to show that depression severity in MDD patients, was significantly associated with functional impairment, specifically absenteeism and presenteeism [36], and productivity loss [37], research regarding the consequences of resistance to MDD therapies in workplace and functional impairment is now emerging.

TRD patients when compared to the general population, were 16 times more likely to have visited an HCP, and four times more likely to have visited a general/family practitioner in the past six months. In addition, TRD patients also had over twice the rate of hospitalizations and ER visits. This is in line with the previous findings wherein TRD patients were associated with multiple negative health outcomes, including increased hospitalization and ER visits [38]. Research studies from Brazil [25], and US [22], also indicated that HRU was significantly increased in the TRD patients when compared to nTRD patients. Furthermore, globally, TRD patients had on average three times more medical visits annually relative to the general population [19].

Hospitalization costs in TRD appeared to be the largest driver of overall economic burden and increased disproportionately to other costs with increasing levels of treatment resistance [35]. Most of the studies showed that higher indirect costs related to absenteeism and productivity loss were associated with greater levels of treatment resistance but the limited data corroborating work productivity and activity impairment in TRD compared to nTRD underscore the importance of our results [23]. Hence, the significantly lower HRQoL, greater work productivity and activity impairment, and increased HRU in TRD compared to nTRD and general population, show that TRD represents a key unmet need.

The enormous impact and humanistic and economic burden of TRD and lack of a consistent definition of TRD suggests the need for a paradigm shift in the approach to care of these patients [12, 14]. In this context, Malhi et al. notes that a more accurate diagnosis of TRD is critical since it is currently viewed as [a] “longitudinal” disease while its diagnostic systems operate “cross-sectionally” [13]. According to Rush et al. [14], TRD is similarly associated with an “acute illness model” representing only a subset of the broader affected patient group with stages of resistance to non-pharmacotherapeutic therapy as well. Disease management of the TRD patient and those in the more comprehensive group would include a full review of the psychiatric and other diagnoses as well as treatment history such as dose, duration, and adherence. Monitoring the patient’s health status and comorbidities, coupled with an effective holistic treatment strategy targeting symptom control and functional improvement may provide a more heuristic approach to alleviating symptoms [14]. Regardless of the breadth of the definition, the TRD patient is in need of multi-dimensional care that consist of pharmacotherapeutic, psychotherapeutic, neuromodulation techniques and/or lifestyle intervention [12–14].

### Limitations

Data from the NHWS are self-reported and cross-sectional. Thus, there is no way to independently verify the variables reported (e.g., diagnosis, medications taken) via some other data source (electronic medical records, physician reports, etc.). However, the survey represents a low-stakes situation and does not present any incentive to purposely misrepresent one’s responses. Also, given the cross-sectional design of the study, statements of causality cannot be made from the results, and temporal trends in the relationships between study variables cannot be ascertained. Additionally, some types of variables (e.g., stopping and starting of medication) could not be assessed because the data were retrospective in nature.

We note that although the TRD construct is complicated, our attempt to identify cases using Committee for Medicinal Products for Human Use (CHMP) guidelines [15] is limited by the availability of data. For example, we cannot determine if TRD cases were due to medical or psychiatric comorbidities, examine TRD subtypes, or account for use of other augmentation therapies. However, the current study is likely to better reflect the TRD population compared to previous study that did not assess the treatment history of TRD population [20], since we used three separate measures of patient identification that included the use of two current chronic prescription medications for depression, the use of a MAOI or RIMA therapy, or a patient-reported response to their current therapy change due to a non-response in their previous therapy.

## Conclusion

In conclusion, TRD patients had significantly lower HRQoL, greater WPAI, and increased HRU as compared with nTRD and general population. The study findings suggest a substantial unmet need exists among TRD patients in Europe. Alleviating health-related and economic burdens in these patients, better TRD management is warranted that includes a multi-pronged approach of improved diagnosis, comprehensive evaluation of environmental and developmental factors driving TRD, and development of specific targeted treatment strategies encompassing pharmacological, psychological, device-based, and/or nutritional therapies.

## Additional files

Additional file 1: **Table S1.** Adjusted mean values for HRQoL among respondents with TRD or nTRD compared to the general population. (DOCX 20 kb)

Additional file 2: **Table S2.** Adjusted mean values for WPAI among respondents with TRD or nTRD compared to the general population. (DOCX 16 kb)

Additional file 3: **Table S3.** Adjusted mean values for HRU use among respondents with TRD or nTRD compared to the general population. (DOCX 16 kb)

Additional file 4: **Figure S1.** Percent of patients with Major Depressive Disorder (MDD) at risk for treatment-resistant depression (TRD). *EUROPE* five European countries, *nTRD* non-treatment-resistant depression, *TRD* treatment-resistant depression, *UK* United Kingdom (TIF 267 kb)

## Data Availability

Anonymized data used and/or analyzed during the current study are available from the corresponding author on reasonable request.

## Abbreviations

BMI: Body mass index
CI: Confidence intervals
EQ-5D-5 L: EuroQol-5 Dimensions 5-levels
EQ-VAS: Euroqol visual analogue scale
ER: Emergency room
HCP: Healthcare professional
HRQoL: Health-related quality of life
HRU: Healthcare resource utilization
MAOI: Monoamine Oxidase Inhibitor
MCS: Mental component summary
MDD: Major depressive disorder
NHWS: National Health and Wellness Survey
nTRD: Non-TRD
OR: Odds ratios
PaCeR: Patient-centered research program
PCS: Physical component summary
PHQ-9: Patient Health Questionnaire
RIMA: Reversible Inhibitor of Monoamine Oxidase
RR: Relative risks
SD: Standard deviation
SF-12v2: Short Form Survey Instrument version 2
TRD: Treatment-resistant depression
WPAI-GH: Work Productivity and Activity Impairment-General Health
