# Supplementary material for: The humanistic and economic burden of treatment-resistant depression in Europe: a cross-sectional study
Source: BMC Psychiatry. 2019 Aug 7;19:247. doi: 10.1186/s12888-019-2222-4 (PMC6686569; doi:10.1186/s12888-019-2222-4)
Supplement: Supplementary file 1 — Table S1. Adjusted mean values for HRQoL among respondents with TRD or nTRD compared to the general population. (DOCX 20 kb) [file 12888_2019_2222_MOESM1_ESM.docx]

**Table S1** Adjusted mean values for HRQoL among TRD, nTRD respondents in the general population

| **Parameter** | **Adjusted Means (SE)^a^** | | | | | |
| --- | --- | --- | --- | --- | --- | --- |
|  | **France** | **Germany** | **Italy** | **Spain** | **UK** | **EUROPE** |
| **EQ-5D-5L** | | | | | | |
| TRD | 0.446 (0.023) | 0.653 (0.012) | 0.747 (0.015) | 0.542 (0.020) | 0.374 (0.013) | 0.513 (0.007) |
| nTRD | 0.590 (0.010) | 0.753 (0.006) | 0.806 (0.007) | 0.716 (0.012) | 0.569 (0.007) | 0.669 (0.004) |
| General population | 0.787 (0.004) | 0.879 (0.003) | 0.896 (0.003) | 0.887 (0.004) | 0.777 (0.005) | 0.842 (0.002) |
| **EQ-VAS** | | | | | | |
| TRD | 47.549 (2.276) | 49.761 (1.592) | 54.335 (2.663) | 48.577 (2.533) | 44.635 (1.239) | 47.637 (0.796) |
| nTRD | 59.530 (1.004) | 57.905 (0.823) | 61.775 (1.251) | 60.372 (1.473) | 56.216 (0.705) | 58.353 (0.414) |
| General population | 73.109 (0.383) | 76.335 (0.421) | 76.717 (0.453) | 77.421 (0.544) | 72.685 (0.439) | 75.077 (0.194) |
| **MCS** | | | | | | |
| TRD | 29.900 (0.990) | 32.033 (0.696) | 31.973 (1.210) | 35.326 (1.041) | 29.284 (0.518) | 30.233 (0.344) |
| nTRD | 35.191 (0.437) | 37.485 (0.360) | 36.706 (0.569) | 39.277 (0.605) | 36.352 (0.295) | 36.227 (0.179) |
| General population | 46.661 (0.167) | 50.237 (0.184) | 46.767 (0.206) | 49.396 (0.224) | 49.182 (0.184) | 48.378 (0.084) |
| **PCS** | | | | | | |
| TRD | 45.610 (0.847) | 44.356 (0.668) | 47.005 (1.009) | 43.246 (0.903) | 43.070 (0.546) | 44.350 (0.318) |
| nTRD | 48.083 (0.374) | 45.973 (0.345) | 48.325 (0.474) | 46.896 (0.525) | 46.490 (0.311) | 47.204 (0.166) |
| General population | 49.562 (0.143) | 49.143 (0.177) | 50.602 (0.172) | 51.273 (0.194) | 48.382 (0.194) | 49.711 (0.078) |
| **SF-12 Bodily Pain** | | | | | | |
| TRD | 40.763 (1.033) | 40.249 (0.800) | 41.327 (1.242) | 38.697 (1.116) | 39.623 (0.612) | 40.072 (0.378) |
| nTRD | 43.235 (0.456) | 42.900 (0.414) | 44.050 (0.584) | 44.347 (0.649) | 43.723 (0.348) | 43.701 (0.197) |
| General population | 47.802 (0.174) | 48.854 (0.212) | 48.881 (0.211) | 51.000 (0.240) | 48.162 (0.217) | 48.843 (0.092) |
| **SF-12 General Health** | | | | | | |
| TRD | 39.909 (0.821) | 37.603 (0.657) | 39.082 (1.174) | 38.467 (0.982) | 36.444 (0.575) | 37.246 (0.330) |
| nTRD | 43.712 (0.362) | 41.605 (0.339) | 42.725 (0.551) | 42.690 (0.571) | 41.484 (0.327) | 42.001 (0.172) |
| General population | 48.911 (0.138) | 48.511 (0.174) | 48.343 (0.200) | 49.486 (0.211) | 48.291 (0.204) | 48.660 (0.081) |
| **SF-12 Mental Health** | | | | | | |
| TRD | 31.684 (0.973) | 36.068 (0.669) | 35.696 (1.207) | 37.557 (1.027) | 32.212 (0.537) | 33.703 (0.341) |
| nTRD | 37.429 (0.429) | 40.814 (0.346) | 39.604 (0.567) | 41.281 (0.597) | 38.254 (0.305) | 39.011 (0.177) |
| General population | 48.107 (0.164) | 50.692 (0.177) | 48.748 (0.205) | 50.811 (0.221) | 49.358 (0.190) | 49.483 (0.083) |
| **SF-12 Physical Functioning** | | | | | | |
| TRD | 43.571 (0.901) | 44.723 (0.659) | 47.698 (1.068) | 43.172 (0.980) | 41.191 (0.566) | 43.582 (0.330) |
| nTRD | 46.993 (0.397) | 46.766 (0.340) | 48.806 (0.502) | 46.953 (0.570) | 45.431 (0.322) | 46.923 (0.172) |
| General population | 50.183 (0.152) | 50.937 (0.174) | 51.802 (0.182) | 51.880 (0.211) | 48.947 (0.201) | 50.686 (0.081) |
| **SF-12 Role Emotional** | | | | | | |
| TRD | 31.772 (1.151) | 29.683 (0.808) | 31.523 (1.391) | 31.941 (1.257) | 28.758 (0.646) | 29.317 (0.407) |
| nTRD | 35.655 (0.508) | 35.034 (0.418) | 35.987 (0.653) | 37.317 (0.731) | 36.472 (0.367) | 35.453 (0.212) |
| General population | 45.836 (0.194) | 48.462 (0.214) | 45.615 (0.236) | 46.930 (0.270) | 47.708 (0.229) | 46.827 (0.100) |
| **SF-12 Role Physical** | | | | | | |
| TRD | 39.935 (0.955) | 38.514 (0.703) | 41.150 (1.118) | 39.822 (1.029) | 37.719 (0.592) | 38.626 (0.349) |
| nTRD | 43.369 (0.421) | 41.114 (0.363) | 43.189 (0.525) | 42.764 (0.598) | 42.841 (0.337) | 42.530 (0.181) |
| General population | 47.858 (0.161) | 47.925 (0.186) | 48.343 (0.190) | 49.303 (0.221) | 47.855 (0.210) | 48.149 (0.085) |
| **SF-12 Social Functioning** | | | | | | |
| TRD | 34.095 (0.973) | 35.351 (0.701) | 36.184 (1.162) | 35.975 (1.022) | 32.115 (0.568) | 33.250 (0.347) |
| nTRD | 38.769 (0.429) | 39.419 (0.363) | 39.421 (0.546) | 40.492 (0.594) | 38.858 (0.323) | 38.680 (0.181) |
| General population | 47.076 (0.164) | 49.567 (0.186) | 46.337 (0.198) | 49.364 (0.220) | 49.091 (0.201) | 48.212 (0.085) |
| **SF-12 Vitality** | | | | | | |
| TRD | 38.138 (0.999) | 40.586 (0.711) | 41.584 (1.175) | 45.212 (1.078) | 38.057 (0.589) | 39.743 (0.357) |
| nTRD | 42.681 (0.441) | 44.159 (0.367) | 45.222 (0.552) | 47.093 (0.627) | 42.549 (0.335) | 43.866 (0.186) |
| General population | 48.818 (0.168) | 52.167 (0.188) | 51.441 (0.200) | 53.998 (0.232) | 50.085 (0.209) | 51.201 (0.087) |

*EQ-5D-5L* EuroQol-5D, *EQ VAS* EuroQol Visual analogue scale, *MCS* mental component scores, *nTRD* non-treatment resistant depression, *PCS* physical component scores, *SF-12* short form version 12, *TRD* treatment resistant depression, *UK* United Kingdom

^a^ Generalized linear models were used adjusted for sociodemographic and health status variables. All comparisons were *p* < 0.05 with the exception of the following cases where TRD and nTRD patients were not statistically different: Italy – PCS, SF-12 Role Physical, SF-12 Vitality; Spain – SF-12 Vitality
