# Supplementary material for: The humanistic and economic burden of treatment-resistant depression in Europe: a cross-sectional study
Source: BMC Psychiatry. 2019 Aug 7;19:247. doi: 10.1186/s12888-019-2222-4 (PMC6686569; doi:10.1186/s12888-019-2222-4)
Supplement: Supplementary file 2 — Table S2. Adjusted mean values for WPAI among respondents with TRD or nTRD compared to the general population. (DOCX 16 kb) [file 12888_2019_2222_MOESM2_ESM.docx]

**Table S2** Adjusted mean values for WPAI among TRD, nTRD respondents and in the general population

| **Parameter** | **Adjusted Means (SE)^a^** | | | | | |
| --- | --- | --- | --- | --- | --- | --- |
|  | **France** | **Germany** | **Italy** | **Spain** | **UK** | **Europe** |
| **Absenteeism, %** | | | | | | |
| TRD | 30.779 (5.992) ^#^ | 35.912 (5.050) ^*#^ | 18.878 (4.225) ^*#^ | 9.133 (1.853) ^*#^ | 37.761 (4.374) ^*#^ | 25.305 (1.793) ^*#^ |
| nTRD | 21.240 (1.669) ^#^ | 22.285 (1.564) ^#^ | 9.266 (0.983) ^#^ | 14.139 (1.687) ^#^ | 25.622 (1.579) ^#^ | 18.147 (0.630) ^#^ |
| General population | 5.975 (0.242) | 7.583 (0.288) | 4.196 (0.185) | 3.933 (0.205) | 6.589 (0.253) | 5.766 (0.105) |
| **Presenteeism, %** | | | | | | |
| TRD | 41.534 (9.318) ^#^ | 45.592 (7.070) ^#^ | 43.207 (9.678) ^*#^ | 37.456 (7.373) ^#^ | 40.872 (5.088) ^#^ | 41.597 (3.150) ^*#^ |
| nTRD | 30.477 (2.547) ^#^ | 32.000 (2.279) ^#^ | 37.248 (3.759) ^#^ | 35.061 (4.104) ^#^ | 35.828 (2.103) ^#^ | 33.570 (1.163) ^#^ |
| General population | 15.405 (0.604) | 14.761 (0.559) | 18.209 (0.748) | 14.490 (0.696) | 17.841 (0.648) | 16.486 (0.288) |
| **Total Work Productivity Impairment, %** | | | | | | |
| TRD | 54.295 (10.418) ^#^ | 59.004 (8.224) ^#^ | 51.933 (11.348) ^*#^ | 41.999 (8.113) ^#^ | 57.849 (6.585) ^#^ | 53.056 (3.695) ^*#^ |
| nTRD | 43.002 (3.304) ^#^ | 43.925 (2.987) ^#^ | 41.234 (4.155) ^#^ | 42.168 (4.837) ^#^ | 47.373 (2.714) ^#^ | 43.316 (1.444) ^#^ |
| General population | 19.411 (0.741) | 19.794 (0.726) | 20.706 (0.844) | 17.117 (0.809) | 20.990 (0.753) | 19.921 (0.342) |
| **Activity Impairment, %** | | | | | | |
| TRD | 57.601 (6.717) ^*#^ | 52.992 (4.546) ^#^ | 52.911 (7.791) ^*#^ | 50.409 (6.577) ^#^ | 61.842 (3.954) ^*#^ | 55.884 (2.330) ^*#^ |
| nTRD | 42.414 (2.186) ^#^ | 44.566 (1.987) ^#^ | 42.466 (2.954) ^#^ | 37.681 (2.886) ^#^ | 48.104 (1.773) ^#^ | 44.197 (0.967) ^#^ |
| General population | 22.976 (0.454) | 21.919 (0.505) | 22.622 (0.576) | 18.138 (0.516) | 23.934 (0.549) | 22.387 (0.231) |

*nTRD* non-treatment resistant depression, *TRD* treatment resistant depression, *UK* United Kingdom

^a^ Generalized linear models were used adjusted for sociodemographic and health status variables.

^*^ Compared to the nTRD population, values for TRD patients differed at *p* < 0.05

^#^ Compared to the general population, values for TRD and nTRD patients differed at *p* < 0.05
