# Supplementary material for: The humanistic and economic burden of treatment-resistant depression in Europe: a cross-sectional study
Source: BMC Psychiatry. 2019 Aug 7;19:247. doi: 10.1186/s12888-019-2222-4 (PMC6686569; doi:10.1186/s12888-019-2222-4)
Supplement: Supplementary file 3 — Table S3. Adjusted mean values for HRU use among respondents with TRD or nTRD compared to the general population. (DOCX 16 kb) [file 12888_2019_2222_MOESM3_ESM.docx]

**Table S3** Adjusted mean values for HRU use among TRD, nTRD respondents and in the general population^a^

| **Parameter** | **Adjusted Means (SE)^b^** | | | | | |
| --- | --- | --- | --- | --- | --- | --- |
|  | **France** | **Germany** | **Italy** | **Spain** | **UK** | **Europe** |
| **HCP visits** | | | | | | |
| TRD | 16.201 (1.933) ^*#^ | 14.737 (1.300) ^*#^ | 14.864 (2.251) ^*#^ | 12.746 (1.722) ^*#^ | 8.687 (0.589) ^*#^ | 12.993 (0.563) ^*#^ |
| nTRD | 10.365 (0.554) ^#^ | 11.179 (0.520) ^#^ | 8.816 (0.643) ^#^ | 9.078 (0.728) ^#^ | 5.812 (0.235) ^#^ | 8.878 (0.205) ^#^ |
| General population | 4.054 (0.088) | 4.416 (0.111) | 4.087 (0.113) | 3.710 (0.117) | 2.660 (0.069) | 3.768 (0.043) |
| **General/Family Practitioner** | | | | | | |
| TRD | 3.545 (0.462) ^#^ | 3.129 (0.305) ^#^ | 3.358 (0.553) ^#^ | 4.186 (0.605) ^#^ | 3.352 (0.249) ^*#^ | 4.025 (0.191) ^*#^ |
| nTRD | 3.320 (0.196) ^#^ | 3.041 (0.156) ^#^ | 2.951 (0.237) ^#^ | 3.043 (0.268) ^#^ | 2.574 (0.116) ^#^ | 3.246 (0.083) ^#^ |
| General population | 1.658 (0.040) | 1.615 (0.046) | 1.812 (0.055) | 1.476 (0.053) | 0.990 (0.031) | 1.506 (0.019) |
| **Hospitalizations** | | | | | | |
| TRD | 0.412 (0.084) ^*#^ | 0.212 (0.040) ^#^ | 0.213 (0.069) ^#^ | 0.292 (0.084) ^#^ | 0.248 (0.043) ^#^ | 0.237 (0.022) ^*#^ |
| nTRD | 0.201 (0.024) ^#^ | 0.162 (0.018) ^#^ | 0.121 (0.024) ^#^ | 0.218 (0.043) ^#^ | 0.211 (0.021) ^#^ | 0.172 (0.010) ^#^ |
| General population | 0.107 (0.006) | 0.101 (0.007) | 0.057 (0.006) | 0.067 (0.007) | 0.160 (0.009) | 0.106 (0.003) |
| **ER visits** | | | | | | |
| TRD | 0.394 (0.088) ^#^ | 0.283 (0.048) ^#^ | 0.338 (0.091) ^#^ | 1.374 (0.236) ^*#^ | 0.613 (0.073) ^*#^ | 0.513 (0.038) ^*#^ |
| nTRD | 0.236 (0.028) ^#^ | 0.196 (0.021) ^#^ | 0.277 (0.039) ^#^ | 0.801 (0.094) ^#^ | 0.339 (0.027) ^#^ | 0.320 (0.015) ^#^ |
| General population | 0.120 (0.007) | 0.110 (0.008) | 0.105 (0.008) | 0.290 (0.017) | 0.195 (0.010) | 0.162 (0.004) |

*ER* emergency visits, *HCP* healthcare professional, *nTRD* non-treatment resistant depression, *TRD* treatment resistant depression, *UK* United Kingdom

^a^ Total visits and hospitalizations in the past 6 months.

^b^ Generalized linear models were used adjusted for sociodemographic and health status variables.

^*^ Compared to the nTRD population, values for TRD patients differed at *p* < 0.05

^#^ Compared to the general population, values for TRD and nTRD patients differed at *p* < 0.05
