# Supplementary figures and images for: The humanistic and economic burden of treatment-resistant depression in Europe: a cross-sectional study
Source: BMC Psychiatry. 2019 Aug 7;19:247. doi: 10.1186/s12888-019-2222-4 (PMC6686569; doi:10.1186/s12888-019-2222-4)

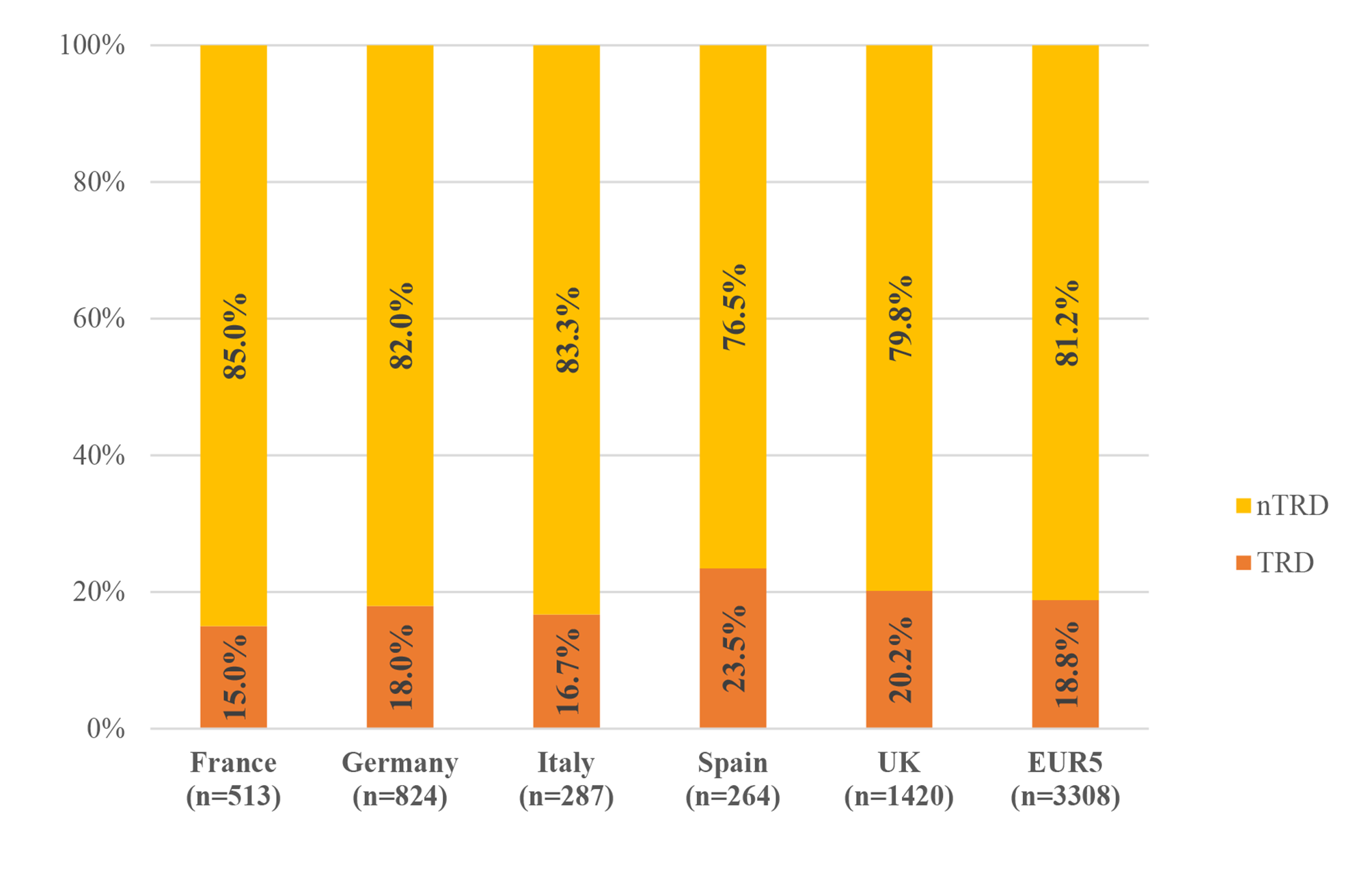

Supplement: Supplementary file 4 — Figure S1. Percent of patients with Major Depressive Disorder (MDD) at risk for treatment-resistant depression (TRD). EUROPE five European countries, nTRD non-treatment-resistant depression, TRD treatment-resistant depression, UK United Kingdom (TIF 267 kb) [file 12888_2019_2222_MOESM4_ESM.tif]
